# Revisiting molecular adsorption: unconventional uptake of polymer chains from solution into sub-nanoporous media†

**DOI:** 10.1039/d1sc03770f

**Published:** 2021-08-18

**Authors:** Noriyoshi Oe, Nobuhiko Hosono, Takashi Uemura

**Affiliations:** Department of Advanced Materials Science, Graduate School of Frontier Sciences, The University of Tokyo 5-1-5 Kashiwanoha, Kashiwa Chiba 277-8561 Japan nhosono@g.ecc.u-tokyo.ac.jp uemurat@g.ecc.u-tokyo.ac.jp; Department of Applied Chemistry, Graduate School of Engineering, The University of Tokyo 7-3-1 Hongo, Bunkyo-ku Tokyo 113-8656 Japan

## Abstract

Adsorption of polymers from the solution phase has been extensively studied to cope with many demands not only for separation technologies, but also for the development of coatings, adhesives, and biocompatible materials. Most studies hitherto focus on adsorption on flat surfaces and mesoporous adsorbents with open frameworks, plausibly because of the preconceived notion that it is unlikely for polymers to enter a pore with a diameter that is smaller than the gyration diameter of the polymer in solution; therefore, sub-nanoporous materials are rarely considered as a polymer adsorption medium. Here we report that polyethylene glycols (PEGs) are adsorbed into sub-nanometer one-dimensional (1D) pores of metal–organic frameworks (MOFs) from various solvents. Isothermal adsorption experiments reveal a unique solvent dependence, which is explained by the balance between polymer solvation propensity for each solvent and enthalpic contributions that compensate for potential entropic losses from uncoiling upon pore admission. In addition, adsorption kinetics identify a peculiar molecular weight (MW) dependence. While short PEGs are adsorbed faster than long ones in single-component adsorption experiments, the opposite trend was observed in double-component competitive experiments. A two-step insertion process consisting of (1) an enthalpy-driven recognition step followed by (2) diffusion regulated infiltration in the restricted 1D channels explains the intriguing selectivity of polymer uptake. Furthermore, liquid chromatography using the MOFs as the stationary phase resulted in significant PEG retention that depends on the MW and temperature. This study provides further insights into the mechanism and thermodynamics behind the present polymer adsorption system, rendering it as a promising method for polymer analysis and separation.

## Abstract 2

Self-entangled large polymeric coils in solution can go into sub-nanometer pores by spontaneous uncoiling, which has been considered unfavorable in conventional molecular adsorption models.

## Graphical abstract

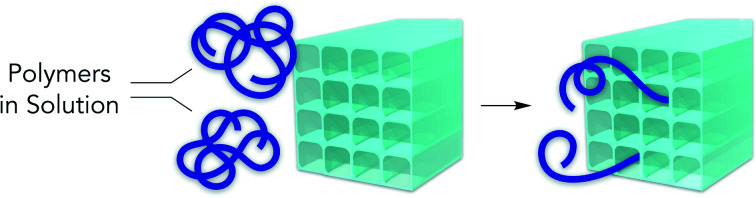

## Introduction

Adsorption of polymers from solution on solid surfaces is a ubiquitous phenomenon that has been studied extensively due to practical needs in a variety of technological aspects, not only for separation and purification methods, but also for the development of coatings and adhesive materials.^1^ Furthermore, adsorption of biopolymers plays a pivotal role in the sensing and signaling functions of biological systems, which forms the basis of biocompatible materials development.^2,3^ The molecular mechanisms of polymer adsorption on flat surfaces have been carefully investigated using inorganic substrates, and many experimental and theoretical studies showed that the interfacial conformation of the polymers as well as interactions between the polymer chains and the substrates fundamentally dictate the adsorption behavior.^4–7^ By contrast, polymer adsorption into porous materials is not clearly understood because many complex matters are involved, including a strong dependence on the relative size between the pore and polymer adsorbates that may range over nanometer to micrometer scales.

Porous materials such as porous carbons, silicas, and zeolites are capable of adsorbing polymers in their voids *via* physisorption from a solution phase. For example, solution-phase adsorptions of polystyrene^8,9^ and polyethylene oxide (PEO)^10–14^ with molecular weight (MW) greater than ∼5 kg mol^−1^ into common porous adsorbents such as activated carbon (pore diameter, *d* ∼ 1.8 nm),^14^ silica (*d* = 7–100 nm),^8–12^ and FAU-type zeolite (*d* = 1.6 nm)^13^ were studied in order to understand the adsorption thermodynamics, kinetics, diffusion, and confinement effects on the adsorbed polymers. In such traditional systems, porous adsorbents that contain pores comparable to or larger than the hydrodynamic diameter of polymer adsorbates have been commonly used because potential void accessibility is considered a prerequisite for polymer admission.^15^ Hence, porous materials that have sub-nanometer pores have rarely been used as the host in past studies, which may be due to the previously held perception that polymers may undergo a tremendous entropic penalty to achieve elongated conformations when entering such nanopores.^16^ As a rare example, PEO adsorption into sub-nanoporous FAU-type (*d* = 0.74 nm) and MFI-type (*d* = 0.55 nm) zeolites from the aqueous phase was shown to significantly slow polymer diffusion by approximately seven orders of magnitude lower than that of the bulk aqueous phase.^17^ However, the mechanism of such polymer insertion into sub-nanoporous solids still remains unclear since rigorous discussion of the results is not yet possible because of the limited data presently available. As an apparently conflicting notion with conventional polymer adsorption arises, we envision that the hitherto unknown problem of how such large polymer coils can diffuse and settle into sub-nanometer pores from the solution phase is paramount.

Metal–organic frameworks (MOFs) have emerged as a new class of porous materials that allow precise tuning of pore sizes, structures, dimensions, and surface functionalities that cannot be attained with conventional porous materials.^18–21^ Owing to such a high degree of designability, MOFs have attracted considerable interest as a functional adsorbent for the separation and storage of gases and small molecules.^22–26^ In the last decade, it was demonstrated that MOFs can also accommodate polymers in their pores, even with pores that are sub-nanometer in size.^27–30^ To incorporate polymers into such small spaces, two methods have been developed: *in situ* polymerization of monomers in the pores^31–34^ and direct insertion of already formed polymers.^35–42^ By exploiting the latter method, many polymer species have been inserted into MOF nanopores to investigate the resulting MOF/polymer composites in which the polymers exhibit unique properties (*e.g.*, thermal and electronic properties^35,39^ and enhanced chemical stability^42,43^) that largely deviate from those in their original state. Most recently, this polymer insertion phenomenon has been employed to discriminate between different polymer structures.^44–48^ In this manner, MOFs recognize minute differences in polymer architectures upon an insertion event, thus allowing precise polymer separation.^47,48^ However, a full understanding of the polymer insertion mechanism remains incomplete despite such prospective applications in the realm of materials science.

**Fig. 1:**
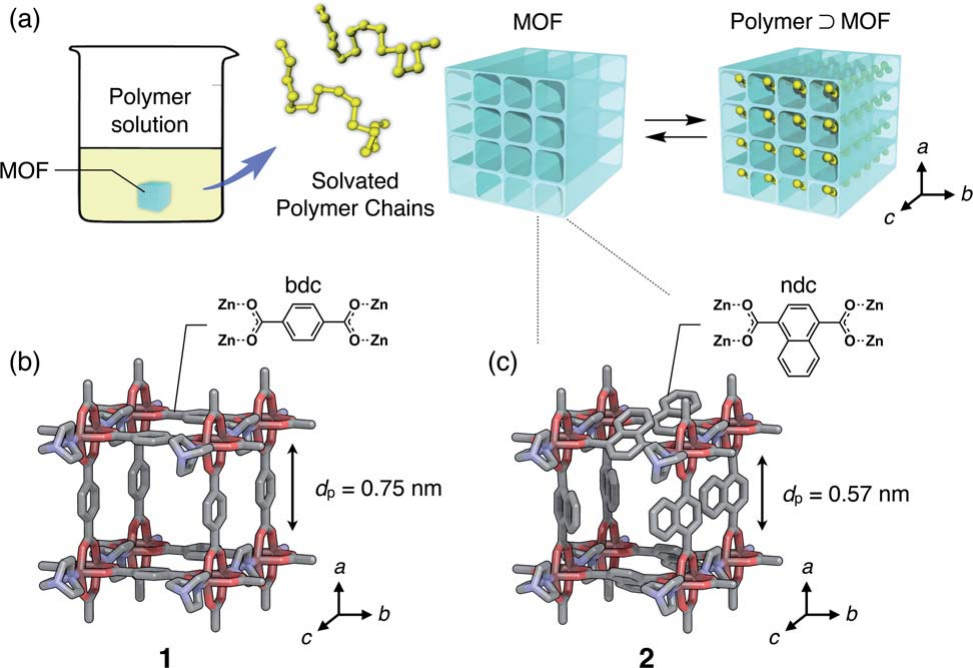
(a) Schematic illustration of polymer insertion into a MOF, which undergoes a complexation equilibrium in the solution phase. Channel structures of (b) [Zn_2_(bdc)_2_ted]_*n*_ (**1**) and (c) [Zn_2_(ndc)_2_ted]_*n*_ (**2**); *d*_p_ denotes the aperture size of the channel. The direction of the 1D channel open for polymer insertion corresponds to the *c*-axis of the MOFs.

In this work, we present the first study of polymer adsorption into sub-nanoporous MOFs from solution phase and report the discovery of unprecedented adsorption behavior that originates from a characteristic two-step guest admission process and restricted diffusion in the channel. We chose polyethylene glycol (PEG), as it is a well-studied polymer that infiltrates many MOFs in the neat phase.^35–37^ In the present work, we demonstrate that even PEGs in the solution phase are spontaneously inserted into one-dimensional (1D) sub-nanochannels of MOFs (Fig. 1). Interestingly, atypical MW selectivity for PEG adsorption was observed in double-component competitive adsorption experiments using two PEGs with different MW, which never happens in conventional meso- and macroporous systems.

Isothermal adsorption experiments for PEGs with various MWs ranging from 0.2 to 20 kg mol^−1^ (Table S1†) were carried out using two isostructural MOFs, [Zn_2_(bdc)_2_ted]_*n*_ (**1**) and [Zn_2_(ndc)_2_ted]_*n*_ (**2**) (bdc = 1,4-benzenedicarboxylate, ndc = 1,4-naphthalenedicarboxylate, ted = triethylenediamine), with sub-nanometer diameters of 0.75 nm and 0.57 nm, respectively (Fig. 1b and c).^49,50^**1** and **2** have a typical pillared-layer structure forming quasi-1D pores along the *c*-axis. Both MOFs showed significant adsorption of the PEGs, in which a remarkable solvent effect was observed. Since the PEG chains should displace the solvent molecules initially present in the pores, the balance of PEG/MOF and solvent/MOF affinities play important roles in the insertion event. Upon insertion, the PEG chains uncoil to attain an elongated conformation in the pores due to the restricted pore size that is comparable to the chain thickness (0.37 nm); therefore, it is envisioned that the system gains sufficient adsorption enthalpy in exchange for the potential entropic loss of uncoiling and disadvantages associated with desolvation upon insertion. Insertion was thereby dependent on the MW of PEGs. Owing to the 1D channel structure of the MOFs, exchange of the guest polymers in the adsorption equilibrium occurs in a highly regulated fashion. Balance of those factors dictating overall insertion dynamics results in peculiar MW selectivity for competitive adsorption that results in faster insertion of larger MW PEGs compared to smaller ones.

The discovery of unconventional polymer uptake driven by chain insertion into MOFs enabled the development of a new class of liquid chromatography for polymer analysis, whereby the MOFs can be used as a versatile stationary phase. This in turn allows us to further understand the thermodynamic factors of the present polymer insertion system. The MOF-column liquid chromatography system shows significant PEG retention that depends on the MW and temperature. This chromatographic method revealed the apparent insertion enthalpies of PEGs exhibiting a linear increase with MW. The present system facilitates polymer separation in an unprecedented enthalpy-driven process of insertion, providing a new class of interaction chromatography (IC) for polymers.^51–55^

## Results and discussion

### Equilibrated adsorption in solution phase

**Fig. 2:**
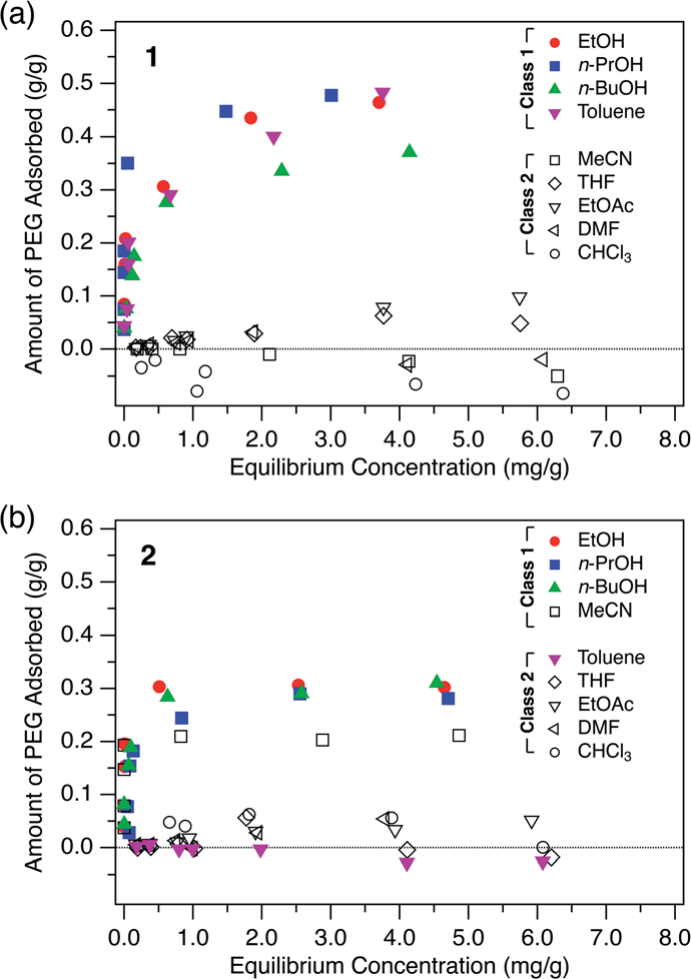
Adsorption isotherms of PEG 2k measured in the given solvents for (a) **1** and (b) **2** at 40 °C.

To investigate the general adsorption capability of the MOFs, adsorption isotherms of PEG were measured for both **1** and **2** using nine typical solvents: ethanol (EtOH), 1-propanol (*n*-PrOH), 1-butanol (*n*-BuOH), toluene, acetonitrile (MeCN), THF, ethyl acetate (EtOAc), DMF, and chloroform (CHCl_3_) at 40 °C (Fig. 2). **1** and **2** in powder form were synthesized with slight modification according to literature procedures (Fig. S1–S3, see ESI†)^49,50^ and evacuated prior to use. A PEG with MW of 2.0 kg mol^−1^, hereafter referred to as PEG 2k, was used as the representative polymer.

As can be seen in Fig. 2, both **1** and **2** showed significant adsorption of the PEG 2k in certain solvents. Particularly, **1** showed great affinity to PEG exclusively in alcohols and toluene. Here we classify the solvents into Class 1 and Class 2, in which the MOF shows good and poor PEG adsorption, respectively. For **1**, alcohols and toluene were categorized into Class 1 (Fig. 2a). In MeCN, THF, EtOAc, DMF, and CHCl_3_, which are Class 2 solvents, **1** did not show significant adsorption even in the higher concentration regime. Likewise, **2** exhibited a similar trend for the solvent dependence of adsorption behavior. Interestingly, however, toluene and MeCN were the only exceptions, displaying an opposite trend to that observed for **1** (Fig. 2b). This intriguing solvent effect implies that solvent molecules play a crucial role in the polymer insertion event. The solvation propensity for PEG, as well as the affinity between the solvent and pore walls, are involved in the description of this thermodynamic system.

**Fig. 3:**
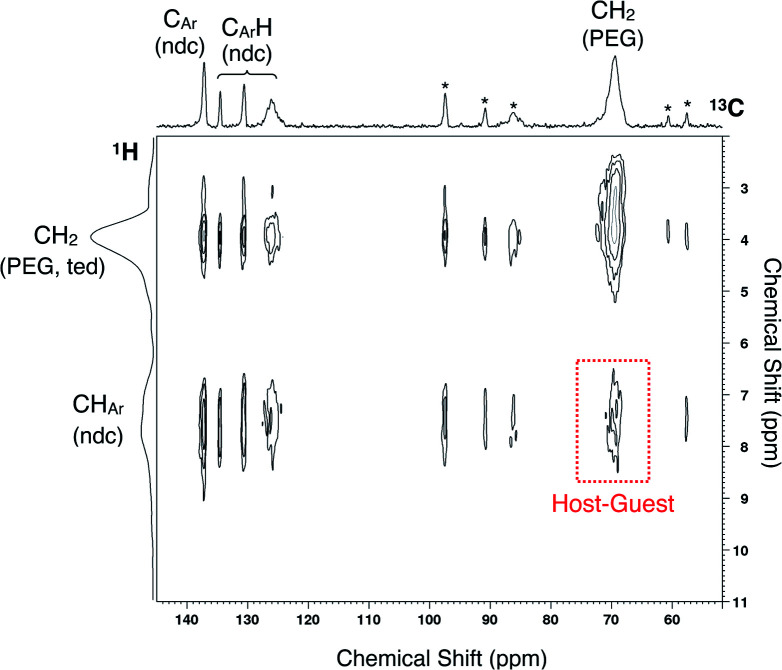
Solid-state 2D ^1^H-^13^C HETCOR NMR spectrum of **2** ⊃ PEG 2k obtained from liquid-phase adsorption using EtOH as the solvent. Asterisks denote spinning sidebands.

To ensure PEG impregnation in the MOF nanopores, 2D ^1^H-^13^C HETCOR NMR spectroscopy was employed to analyze the PEG inserted sample of **2** (**2** ⊃ PEG 2k) obtained from solution phase adsorption using EtOH as the solvent (Fig. 3). The spectrum showed a cross peak between carbons of the ndc ligand in the framework and PEG protons, providing reliable evidence for the occlusion of PEG in individual MOF pores. It should be noted that the radius of gyration (*R*_g_) of PEG 2k in good solvent is approximately 1.5 nm, *i.e.* 3.0 nm in diameter,^56^ which is ∼5 times larger than the pore aperture of **2**. The same solution-phase adsorption experiment using EtOH as the solvent was also performed for PEG 20k, whose *R*_g_ is ∼4 nm (*i.e.*, ∼8 nm in diameter) in good solvent.^57^ The solid-state NMR spectrum showed a similar cross peak at the corresponding position, confirming the accommodation of such a macromolecular guest from the solution phase (Fig. S4†). Hence, uncoiling of the solvated PEG chain is a requisite process upon admission into the pore.

Each adsorption isotherm (Fig. 2) measured in a Class 1 solvent showed a good fit to the Langmuir model, which is expressed by the following formula:
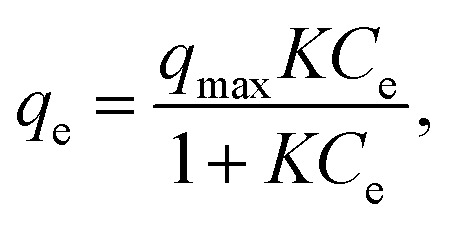
where *q*_e_ is the amount of adsorbed PEG per unit weight of MOF, *C*_e_ is the equilibrium concentration of PEG, *K* is the apparent equilibrium constant, and *q*_max_ is the maximum amount of adsorbed PEG. The *q*_max_ values for Class 1 isotherms are summarized in Table 1. The *q*_max_ for **1** was ∼0.5 g g^−1^ for Class 1 solvents, which corresponds to the potential maximum capacity of **1** previously determined by the direct insertion method using neat PEGs.^35^ Likewise, **2** displays high affinity for PEG in the adsorption isotherm of Class 1 solvents; **2** adsorbs PEG 2k up to *q*_max_ of ∼0.3 g g^−1^, which is comparable to the predetermined maximum capacity of **2**.^46,47^ These findings indicate that PEGs are spontaneously inserted into both types of MOFs in the solution state with progressive replacement of the solvent molecules to be exchanged. At higher concentrations, MOF nanopores become entirely occupied with desolvated PEG molecules. Thus, while being inserted into the nanopores, PEG molecules undergo considerable desolvation in addition to the expected entropic loss for uncoiling, which in turn indicates the presence of an extraordinary enthalpic gain that compensates for such energetic drawbacks.

**Table 1:** Results of Langmuir fits to the adsorption isotherms for PEG 2k at 40 °C

Solvent (class 1)	**1**	**2**
	*q*_max_ (g g^−1^)	*q*_max_ (g g^−1^)
EtOH	0.46	0.30
*n*-PrOH	0.47	0.30
*n*-BuOH	0.38	0.31
Toluene	0.48	—
MeCN	—	0.21

The driving force for insertion can be assessed qualitatively based on the propensity of PEG to dissolve in the solution. Table 2 lists the Hansen solubility parameters of PEG in given solvents.^58,59^*R* denotes the distance between the Hansen parameters of two substance coordinates, namely PEG and the given solvent, in Hansen space. A smaller *R* value denotes that PEG and the solvent are more likely to be compatible with each other. Therefore, the *R* value can be used as an apparent measure of the extent of solvation of PEG molecules in the solution phase. As seen in Table 2, Class 1 solvents for **1** (toluene and alcohols) have larger *R* values than those of Class 2 solvents (CHCl_3_, MeCN, EtOAc, THF, and DMF). This indicates that PEG molecules tend to be intercalated in **1** when the surrounding solvent molecules have poor affinity for the PEG molecules. In other words, PEG insertion appears to be a favorable process in the poor solvents (Class 1) but not in the good solvents (Class 2) as a result of the overall balance among multiple factors (*e.g.*, PEG adsorption energy gains and PEG desolvation energy costs). This is an interesting consequence, since polymers generally adopt a globular and compact conformation in the poor solvent, in which uncoiling upon insertion would be considered a rather difficult process.

**Table 2:** Hansen solubility parameters^58,59^

	*δ*_d_ (MPa^1/2^)	*δ*_p_ (MPa^1/2^)	*δ*_h_ (MPa^1/2^)	*R* (MPa^1/2^)
PEG	17	10.7	8.9	—
Toluene	18.0	1.4	2.0	11.6
EtOH	15.8	8.8	19.4	10.7
*n*-PrOH	16.0	6.8	17.4	9.4
*n*-BuOH	16.0	5.7	15.8	8.6
CHCl_3_	17.8	3.1	5.7	8.3
MeCN	15.3	18.0	6.1	8.0
EtOAc	15.8	5.3	7.2	5.8
THF	16.8	5.7	8.0	5.1
DMF	17.4	13.7	11.3	3.9

In addition, interactions between the MOF and solvent molecules should be taken into account to explain the effect of the solvent. Upon polymer insertion, the solvent molecules initially present in the pore are displaced by PEG and subjected to exchange. Therefore, if the solvent has a higher affinity for the MOF than does PEG, then PEG insertion does not occur. Considering the solvent affinity difference of the MOFs, the exceptional solvent effects observed for **2** can be partly explained (Fig. 2). Since **2** has more aromatic rich side walls compared to **1**, toluene molecules in **2** can be more stabilized than in **1** through possible π–π interactions in the confined space. Therefore, PEG insertion from a toluene solution into **2** is a more difficult process compared to its insertion into **1** since toluene occupying the pores has to be displaced from **2**, resulting in a larger desorption energy than that of **1**. However, the effect of MeCN on the exceptional adsorption behavior of **2** does not seem to be explained by the MOF-solvent and MOF-polymer interactions, and requires further investigation.

To gain a better understanding of the origin of the solvent dependence, we have to pay attention also to the behavior of solvent molecules confined in the pores. Depending on the pore size, solvent molecules can be forced to adopt a distorted and unfavorable intramolecular geometry that may preclude proper solvent–solvent interactions. Hence, the solvent molecules once liberated from the MOF pores may achieve stabilization by recovering the solvent–solvent interactions. Such enthalpic stabilization of the liberated solvent molecules, which is more pronounced for hydrogen-bonding solvents such as alcohols, also makes the entire insertion process energetically favorable.^60^ Further, entropic contributions, which include the balance between the conformational entropy of the PEG chain and the translational entropy of the solvent, should be considered as well.^61^ While loss of the conformational entropy of polymer chains is expected upon insertion, the translational entropy gain of solvent molecules that are liberated from the MOF pores can be one of the counterbalancing benefits.

As discussed above, unlike gas adsorption, in which adsorbents are fully evacuated before use, polymer adsorption into MOFs from the solution phase should be treated as a competing process with solvent molecules that occupy the pores. Hence, not only polymer/MOF affinities, but also solvent/polymer, solvent/MOF, and solvent–solvent interactions must be considered in order to control and understand the present polymer insertion system.

### Molecular weight dependence

**Fig. 4:**
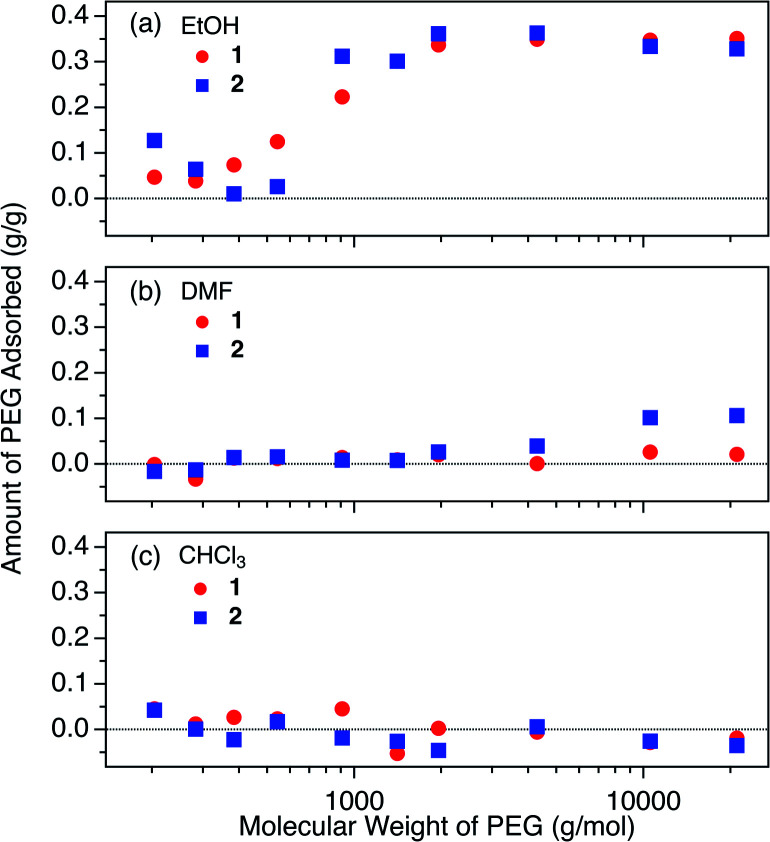
The influence of molecular weight on the adsorbed amount of PEG (2.0 mg g^−1^) in (a) EtOH, (b) DMF, and (c) CHCl_3_ at 40 °C.

We carried out adsorption experiments using PEGs with various MWs of 0.2k–20k (Table S1†), and investigated the influence of MW on the equilibrium adsorption for **1** and **2** at 40 °C in EtOH. The initial PEG concentration was fixed at 2.0 mg g^−1^. The present PEG insertion system exhibited an unconventional MW dependence of the adsorption behavior, suggesting cooperative insertion mechanism that has never been reported for conventional meso- and macroporous systems (Fig. 4).

In EtOH, **1** showed a gradual increase of the uptake with increasing the MW of PEGs above 0.3k (Fig. 4a), and **2** exhibited a similar trend except with a more rapid increase of PEG uptake at 0.4k. These observations indicate that there is a specific MW threshold at which polymer intercalation abruptly occurs. In other words, polymer intercalation into MOFs from the solution phase requires a certain chain length (*i.e.*, number of monomer units constituting the chain) to attain sufficient enthalpy in order to trigger the insertion event. Although it is generally known that polymer MW largely affects adsorption behavior, such cooperativity in polymer adsorption has not previously been reported for polymer adsorption systems of conventional porous materials with open pores larger than the polymer coils.^10–13,62^ This cooperativity can be attributed to the unique way that polymers enter into the 1D pores of MOFs **1** and **2** at the onset of the insertion process, whereby the polymer chains are always inserted from their termini. At the interface between the MOF and polymer solution, a polymer terminus undergoes a dynamic insertion/rejection process controlled by complex equilibria. During this dynamic process, the polymer length (*i.e.*, MW) is recognized by the MOF pore entrance from a combination of energy balances among adsorption, desolvation, and solvent exchange. The origin of this cooperative insertion effect appears to be different from reported examples of cooperative molecular adsorption mechanisms for some porous materials.^63,64^ It may be rather explained by the cooperative binding model that has been applied to protein binding on solid supports.^65,66^

Considering the balance of entropic factors of this insertion system also provides insights into the mechanism of the MW dependence. The conformational entropy loss of polymer chains and the translational entropy gain of the solvent molecules pushed out from the MOF pores are counterbalancing factors for the insertion, and the two are not always comparable. The similar MW effect can be seen in protein folding phenomenon.^61^ The denatured polypeptide chain with a certain threshold length (*i.e.* number of residues) is spontaneously folded into the native structure in water. At this threshold length, the translational entropy gain for the solvent water molecules becomes high enough to compensate the conformational entropy loss of the polypeptide chain, making the folding process energetically favorable. To gain a fuller understanding of the MW dependence of the present insertion system, identifying such enthalpic and entropic contributions *in silico* would be an important task for the future.

The MW dependence of PEG uptake differed in other solvents (*i.e.*, DMF and CHCl_3_, Fig. 4b, c). In DMF, **2** displayed a weak affinity for PEGs with MW above 2k, whereas none was observed for **1**. In CHCl_3_, both **1** and **2** showed no appreciable adsorption, even for the high MW PEG of 20k. The solvent effects observed for DMF and CHCl_3_ for the higher MW PEGs diverge from that expected from the Hansen solubility parameters, as the *R* value between PEG and CHCl_3_ is larger than that between PEG and DMF (Table 2). This can be explained by the effect of MW on the Hansen parameter of PEG, which causes a substantial decrease of *δ*_d_ and *δ*_p_, and an increase of *δ*_h_ for higher MW.^67^

### Polymer insertion kinetics

How polymers diffuse into 1D MOF channels is important to consider as a means to understand the overall mechanism of the polymer insertion phenomenon. However, to the best of our knowledge, polymer insertion kinetics into MOFs from the solution phase has never been investigated to date. Polymer insertion kinetics into sub-nanometer pores in particular – whose size is close to the polymer chain thickness – has been considered difficult from common kinetic and thermodynamic aspects, and therefore has scarcely been reported except for an example of PEG adsorption in zeolites in aqueous media.^17^

**Fig. 5:**
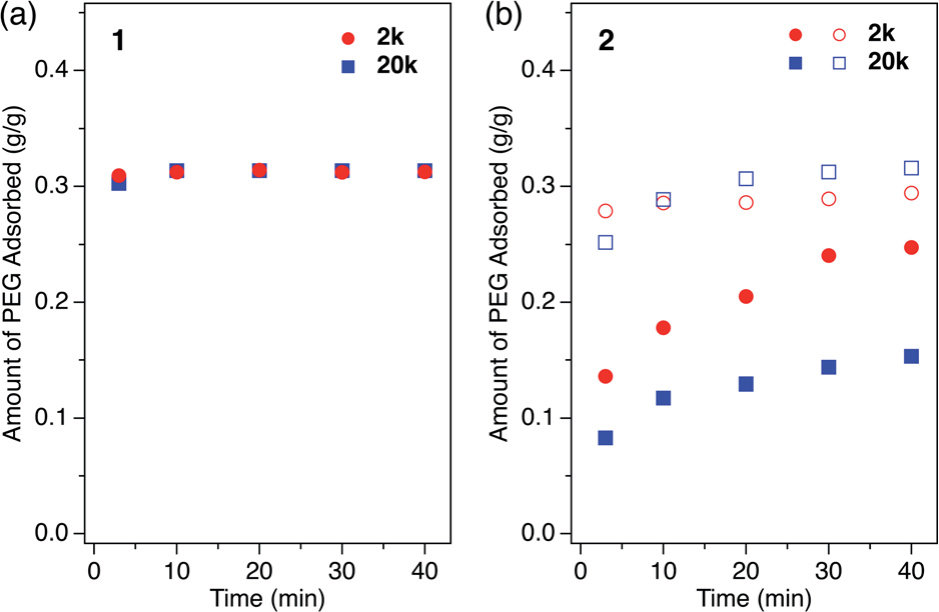
Kinetic plot for single-component PEG insertion into (a) **1** and (b) **2** in EtOH, measured using PEGs of (red circle) 2k and (blue square) 20k at (closed symbol) 40 °C and (open symbol) 60 °C. The initial concentration of PEG was 1.2 mg g^−1^ for all samples.

We investigated the PEG insertion kinetics in **1** and **2** for solution phase and isothermal conditions. The time evolution of PEG adsorption for each MOF was measured using PEG 2k and 20k in EtOH (Fig. 5). The insertion kinetics for both 2k and 20k into **1** was very fast and reached the fully adsorbed state at the given concentration (2.0 mg mL^−1^) within 3 min at 40 °C (Fig. 5a). On the other hand, **2** displayed slower PEG insertion kinetics under the same conditions, which took more than 40 min to reach the fully adsorbed state (Fig. 5b). This is reasonable since **2** has a smaller pore size compared to **1**. The smaller pore size of **2** results in less frequent entry of the chain termini into the MOF, as well as impeded mobility in the channel when compared to the larger one. Similar pore size effects have also been reported for polymer adsorption in other porous materials.^10,12,17^ For insertion of PEG into **2**, an effect of the MW on the insertion rate was observed (Fig. 5b), with the shorter PEG 2k exhibiting faster insertion compared to the longer PEG 20k. This is reasonable because the polymer diffusion rate in the porous media generally depends on the MW.^9,13,68–70^ Several experimental and theoretical examples of polymer adsorption into mesoporous materials showed negative exponent laws between the polymer MW and its effective diffusion coefficient, *D*_eff_, in the pore.^13,68–70^ At an elevated temperature of 60 °C, the insertion of both 2k and 20k PEGs into **2** became faster, reaching the fully adsorbed state within 10 min and 20 min, respectively (Fig. 5b).

Although it was not possible to perform a kinetic analysis of **1** due to the extremely fast insertion rate, the kinetic plot for **2** at 40 °C was analyzed and fit well to a pseudo-second-order model that is expressed by the following formula:
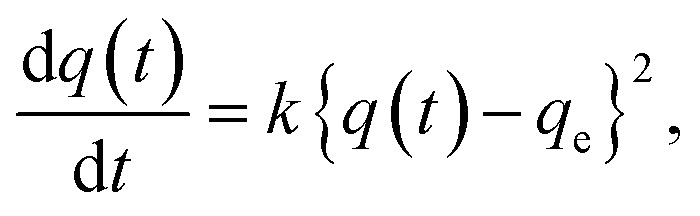
where *k* is the pseudo-second-order rate constant, *q*(*t*) is the amount of adsorbed PEG after *t*, and *q*_e_ is the maximum amount of adsorbed PEG at a given concentration at the equilibrated state.^71^ Fitting analysis provided the rate constant, *k* (40 °C), of 0.53 and 0.13 g g^−1^ min^−1^ for PEG 2k and 20k, respectively. Based on the kinetic data and assuming Fickian diffusion, *D*_eff_ of PEG 2k and 20k in the nanochannels of **2** were calculated to be 1.3 × 10^−13^ and 3.1 × 10^−14^ m^2^ s^−1^, respectively (see ESI†), which are approximately three orders of magnitude larger than the values reported for PEGs in an MFI zeolite with a similar pore size of ∼0.55 nm (25 °C in water).^17^ Because the MFI zeolite contains 3D channels, PEG chains threaded through the interconnected pores may interfere with the diffusion of other chains as well as themselves (self-avoiding effect), plausibly causing such suppressed *D*_eff_. On the other hand, for the regular 1D channels in MOF **2**, PEG chains can diffuse and infiltrate the crystallites without such interference, facilitating smooth diffusion.

### Competitive polymer insertion

**Fig. 6:**
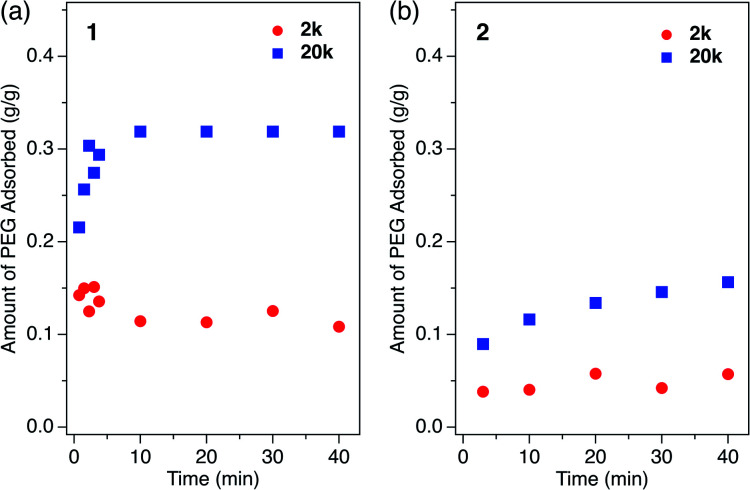
Kinetic plot for double-component competitive PEG insertion into (a) **1** and (b) **2** in EtOH, measured using the mixture of PEG 2k and 20k at 40 °C. The inserted amount of (red circle) 2k and (blue square) 20k are plotted as a function of time. The initial concentration of each PEG was 1.2 mg g^−1^ (2.4 mg g^−1^ total) for all samples.

If polymers with different MW are mixed in the solution phase, the insertion process occurs in a competitive manner. Such competitive insertion among multiple components is of high importance in the scope of separation applications. We measured the double-component competitive insertion kinetics for **1** and **2** with the same method as described earlier for the single-component measurements, except using a solution containing two PEGs (2k and 20k) in EtOH. Interestingly, we found opposing MW dependence of the insertion kinetics in the competitive system (Fig. 6). For both systems **1** and **2**, the longer PEG 20k exhibited faster insertion than PEG 2k when equal amounts of each PEG were present in the mixture.

To the best of our knowledge, such kinetic behavior has never been reported before in a polymer adsorption system, and cannot be explained by the conventional theory of adsorption kinetics developed for solid surfaces and porous materials. Previously reported examples of competitive polymer adsorption on flat substrates demonstrated that the adsorption of shorter chains occurs first due to kinetic advantages, followed by the adsorption of longer chains.^72–75^ Moreover, in typical porous silicas with pore diameters of 30–130 nm, the shorter chains show faster adsorption rates than those of longer competing chains.^11^ In such conventional polymer adsorption systems, the slower adsorption of the longer chains always occurs concomitant with desorption of the pre-adsorbed shorter chains to be exchanged in the adsorption layer, because the longer chains contribute to stabilizing the system with their higher adsorption energy per molecule. Hence, the exchange process is likely to be inhibited when the pore diameter is smaller than the size of coiled polymers in solution.

In MOFs **1** and **2** containing 1D channels with extremely narrow pores that are comparable to the PEG chain thickness of ∼0.37 nm, the PEG chains are no longer allowed to overtake other chains in front of them. Hence, once the longer PEG 20k is inserted, the following insertion of PEG 2k is blocked and prohibited by PEG 20k in front of it, even though the individual diffusion of PEG 2k is actually faster than PEG 20k. In other words, the originally slower diffusion of PEG 20k is the rate-limiting process of the entire competitive insertion. Indeed, in the competitive experiments of both systems **1** and **2**, the kinetic plot of PEG 20k insertion (Fig. 6) is comparable to that observed for the individual insertion experiments at the same temperature (Fig. 5), indicating that the insertion rate of longer PEG 20k is unchanged regardless of the presence of shorter PEG 2k (Fig. S5†). On the other hand, the PEG 2k insertion process was significantly slowed due to the co-adsorption with PEG 20k. It should be noted that PEG 2k that was co-adsorbed with PEG 20k did not desorb from the MOF, and remained in the pores for a long time (two weeks) without being displaced by PEG 20k under these conditions, which underpins our hypothesis that the exchange between long and short PEGs is prohibited due to confinement by the sub-nanometer pores.

**Fig. 7:**
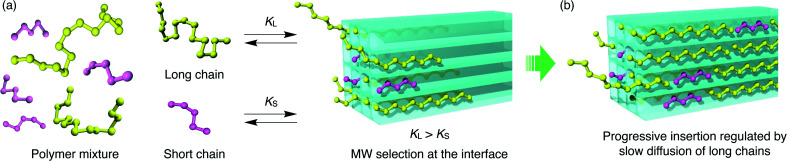
Schematic illustrations of a plausible two-step mechanism for competitive polymer insertion into MOFs. (a) The first step in which longer polymers are selected to be inserted into the MOF under the control of thermodynamic equilibrium. *K*_L_ and *K*_S_ denote equilibrium constants for initial insertion of long and short chains, respectively. (b) The second step that contributes to the kinetics of the insertion process. Diffusion of the longer chains is slow and becomes the rate-limiting process of the entire insertion event.

For the competitive insertion experiments, each PEG concentration was fixed by weight at 1.2 mg g^−1^ (2.4 mg g^−1^ total). Therefore, the mol concentration of PEG 2k is 10 times higher than that of 20k in solution, which would appear as if PEG 2k is much more likely to be inserted preferentially when considering the collision frequency on the MOF surface, although this is not the case in the present MOF-based insertion system. This intriguing phenomenon is explained by the following two-step insertion mechanism (Fig. 7). (1) The first step consists of the interfacial adsorption equilibrium between polymer chains and MOF surfaces (Fig. 7a). At the onset of insertion, each polymer chain undergoes a dynamic insertion/rejection process at the MOF surface under equilibrium, in which each PEG has its own equilibrium constant, *K*, that is correlated with the Gibbs free energy of the first insertion in the MOF nanopores. Since the adsorption enthalpy, Δ*H*, should be more negative for higher MW (*i.e.*, number of repeating units) polymers, longer polymers are preferentially introduced into the MOF nanochannels. (2) The second step is a slow process governed by the intraparticle Fickian diffusion of individual chains, which is mostly controlled by the diffusion rate of larger MW polymers (Fig. 7b). The solvation effect associated with the PEG chains is also dependent on the MW, which can be accounted for in the first step. In general, higher MW PEGs are less soluble than shorter PEGs, which could also facilitate the preferential insertion of higher MW PEGs, as described in the previous section.

### MOF-packed column chromatography

**Fig. 8:**
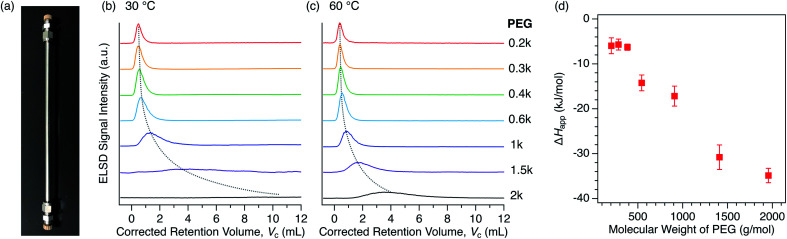
(a) Photograph of the actual **2**-packed column used for HPLC with dimensions of 4 mm I.D. × 250 mm L. HPLC chromatograms for PEGs on the **2**-packed column using DMF as the eluent at (b) 30 °C and (c) 60 °C. The dotted lines are included to guide the eye. (d) MW dependence of Δ*H*_app_ calculated on the basis of temperature dependent retention data for each PEG. See ESI† for the calculations and van't Hoff plots (Fig. S9†).

For the purpose of understanding the insertion thermodynamics as well as demonstrating a potential application involving polymer separation, we performed liquid chromatography using the MOFs as a stationary phase. Examples of MOF-based LC have hitherto been limited for the separation of small molecules,^76–80^ except our recent work aimed at the recognition of polymer termini.^47^ We first packed columns with either **1** or **2** to examine the PEG retention capability using a conventional high-performance liquid chromatography (HPLC) system (Fig. 8a). PEGs with different MW were injected into each packed column using DMF as the eluent. Interestingly, the column packed with **2** exhibited significant retention of the PEGs (Fig. 8b, c), while the column packed with **1** did not show any retention at all (Fig. S6†). This implies that interactions between **1** and PEG are somehow weaker than those between **2** and PEG, ultimately being too weak to allow appreciable retention on the column at the given temperature in DMF. This is in agreement with the difference in affinity between **1** and **2** in DMF, as observed in the static adsorption experiments (Fig. 4b), which showed superior adsorption of **2** to **1**. It was previously reported that PEGs accommodated in Cu-based **2** ([Cu_2_(ndc)_2_ted]_*n*_, which is structurally identical to **2** except for the metal center) are more stable than those in Cu-based **1**, *i.e.*, [Cu_2_(bdc)_2_ted]_*n*_, arising from possible CH-π interactions between the PEG main chain and ndc ligands.^35^ It should be noted that the eluent lends a striking effect to the retention behavior. Class 2 solvents, including DMF, were rather useful for the MOF-column LC analysis of PEGs, whereas the retention in Class 1 solvents is too strong, leading to no PEG elution at all within a reasonable temperature range (Fig. S7†). The MOF-packed columns provided chromatograms with high reproducibility, while also displaying promising stability of the working performance for practical use. In fact, the PXRD pattern of **2** used for the stationary phase of the column showed no change in the structural integrity compared to that of freshly synthesized **2**, even after one year of use with DMF at 80 °C (Fig. S8†).

We further analyzed the PEG retention behavior on the column packed with **2** to gain a better understanding of the thermodynamic aspects of this insertion system. On the **2**-packed column, PEG 0.2k was eluted at the corrected retention volume, *V*_c_ = 0.385 mL, whereas higher MW PEGs were eluted later at *V*_c_ = 0.394, 0.444, 0.546, 0.868, 1.730, and 3.530 mL for 0.3k, 0.4k, 0.6k, 1k, 1.5k, and 2k, respectively, at 60 °C (Fig. 8c, see ESI†). In our previous study, we demonstrated that the end groups of PEG significantly affect the retention behavior. PEG 2k end-capped with a trityl group, which is larger in size than the pore size of **2**, did not show retention on the **2**-packed column at all.^47^ This clearly indicates that polymer insertion is the prevailing mechanism of column retention. It is noteworthy that the present MOF column displays elution behavior that is opposite to that of conventional SEC columns, in which higher MW polymers elute faster than lower MW ones. As can be seen in the chromatograms (Fig. 8c), the higher MW PEGs exhibited considerable broadening of the elution peak, which is attributed to slow exchange occurring at the MOF/solution interface as a dynamic insertion/rejection process. Therefore, this column retention behavior is associated with the thermodynamics of the first insertion step, thus providing a clue for the apparent *K* of the host-guest interfacial equilibrium (Fig. 7a).

The retention behavior of PEGs on the **2**-packed column changed significantly with temperature. At lower temperature of 30 °C, elution peaks for all PEGs were shifted to a larger retention volume, *V*_c_ (Fig. 8b). In addition, the peak shape becomes broader than that measured at 60 °C, suggesting that the interfacial exchange process becomes slower at lower temperature. The temperature dependence of the *V*_c_ allows us to calculate the apparent adsorption enthalpies and entropies, Δ*H*_app_ and Δ*S*_app_, respectively, for each PEG on the basis of the van't Hoff relationship.^51–55^ The van't Hoff plots were successfully obtained based on the chromatograms recorded at multiple temperatures ranging from 30 °C to 75 °C (Fig. S9†), allowing the determination of Δ*H*_app_ and Δ*S*_app_ in terms of the entire PEG molecule (Table 3). The Δ*H*_app_ becomes more negative for higher MW PEGs, suggesting that the interaction-driven mechanism is most likely for the MOF-column LC system. Interestingly, Δ*S*_app_ also becomes negative with increasing MW, which is possibly due to a loss of entropy associated with uncoiling of polymer chains when inserted into the sub-nanometer pores of MOFs. Because of the large increase in Δ*H*_app_ and slower exchange process, PEG 20k required a much higher column temperature of >80 °C, which is outside the control limit of the current analytical system. Considering this for future development, a binary gradient flow using a Class 1 and Class 2 solvent mixture can be a useful strategy to gain full control of column retention behavior over a wide range of polymer MWs and polymer species.

**Table 3:** Δ*H*_app_ and Δ*S*_app_ in terms of PEG chain determined by the van't Hoff plot for HPLC data on the **2**-packed column

PEG	Δ*H*_app_^a^ (kJ mol^−1^)	Δ*S*_app_^a^ (J K^−1^ mol^−1^)
0.2k	−6.0 ± 1.8	−32.3 ± 5.6
0.3k	−5.7 ± 1.2	−31.0 ± 3.8
0.4k	−6.3 ± 0.70	−32.0 ± 2.2
0.6k	−14.2 ± 1.8	−53.9 ± 5.2
1k	−17.2 ± 2.2	−58.9 ± 6.6
1.5k	−30.8 ± 2.7	−94.0 ± 8.1
2k	−34.9 ± 1.6	−100.4 ± 4.7

a See ESI for the calculations and corresponding van't Hoff plots (Fig. S9).

The Δ*H*_app_ exhibited a trend proportional to the MW of PEG above 0.6k (Fig. 8d), as observed for conventional interaction chromatography (IC) of polymers.^51–53^ In IC, Δ*H* increases proportionally to the number of repeating units (*i.e.*, degree of polymerization) of polymers, which is known as Martin's rule.^54,55^ However, this does not promise that the rule is always applicable, as the present MOF-based LC system exhibits a retention mechanism that is based on the insertion of polymer termini, which is fundamentally different from IC systems using conventional stationary phases. Additionally, Table 3 shows another interesting aspect that there is a threshold value of MW (0.6k) where Δ*H*_app_ begins to show negative growth. This is potentially due to the terminal effect. The contribution of terminal hydroxyl groups of the PEGs becomes more prominent for the shorter chains, affecting the affinity balance among solvent, MOF, and PEG with the MW below the threshold. Such terminal effect on the thermodynamic parameters is also observed in IC of polymers.^81^ Further study on this intriguing column retention mechanism will be performed in our group with the aim of developing a new methodological framework for macromolecular recognition and separation.^44^

## Conclusions

In this work, we reported that polymer insertion into sub-nanoporous MOFs spontaneously occurs from the solution phase. Admission of polymer chains that adopt coiled and self-entangled conformations with extremely larger hydrodynamic diameters relative to the pore diameter is possible when the system attains sufficient enthalpy to override potential entropic losses due to uncoiling of the solvated polymer chains. The thermodynamics and kinetics of polymer insertion were investigated using PEGs, which unveiled an enthalpy-driven mechanism associated with a dynamic insertion/rejection process at the solution/MOF interface. Isothermal adsorption analysis revealed a unique solvent dependence of PEG insertion, which is explained by affinity between PEG and the MOFs, affinity between the MOFs and solvents, and the solvation effect.

Intriguing MW dependence was observed for the PEG insertion kinetics. In the single-component insertion experiments, normal behavior with shorter chain insertion occurring faster than longer chain insertion was observed. In stark contrast, the competitive insertion experiments using a binary mixture of different MW PEGs revealed the opposite: longer chain insertion prevails and an apparent reversal in the trend of insertion kinetics occurs. This peculiar MW dependence is explained by a two-step insertion mechanism consisting of (1) MW recognition followed by (2) rate-limiting insertion.

The MOF column chromatography of PEGs showed interaction-based retention that depends on the PEG MW and the column temperature. This feature allowed us to develop a further understanding of the thermodynamics underlying the polymer insertion system, which renders this MOF chromatography as a promising new polymer discrimination method. We believe that these findings will contribute greatly to the development of polymer analysis and separation technologies using MOFs as versatile recognition media.

## Data availability

All data supporting the findings of this study are available within the paper and its ESI.†

## Author contributions

N. O. performed experiments, calculations, and analysis of the experimental data. N. O. and N. H. designed the study and experiments. N. H. and T. U. conceptualized and supervised the project. All authors discussed the results and wrote the manuscript.

## Conflicts of interest

There are no conflicts to declare.

## Supplementary Material

SC-012-D1SC03770F-s001
