# Supplementary material for: Revisiting molecular adsorption: unconventional uptake of polymer chains from solution into sub-nanoporous media
Source: Chem Sci. 2021 Aug 18;12(38):12576–86. doi: 10.1039/d1sc03770f (PMC8494126; doi:10.1039/d1sc03770f)
Supplement: SC-012-D1SC03770F-s001 [file SC-012-D1SC03770F-s001.pdf]

Electronic Supplementary Material (ESI) for Chemical Science.
This journal is © The Royal Society of Chemistry 2021

Electronic Supplementary Information

Revisiting Molecular Adsorption: Unconventional Uptake of Solvated Polymer Chains into Sub-Nanoporous Media

Noriyoshi Oe, Nobuhiko Hosono, Takashi Uemura

Table of Contents

1. **General Instruments** 1
2. **Materials** 2
3. **2D HETCOR NMR Analysis** 2
4. **Adsorption Isotherm Measurements** 3
5. **Adsorption Kinetics Measurements** 3
6. **HPLC Analysis on MOF-Packed Column** 3
7. **General Procedure for Determination of Adsorbed Amount of PEG** 4
8. **Calculation of Effective Diffusion Coefficient** 5
9. **van't Hoff plot** 6
10. **Supporting Table** 6
11. **Supporting Figures** 7
12. **Supporting References** 12

1. General Instruments

^{1}H nuclear magnetic resonance (NMR) spectra were recorded using a JEOL model ECS-400 spectrometer operating at 400 MHz. Powder X-ray diffraction (PXRD) data were recorded on a Rigaku model SmartLab X-ray diffractometer using Cu Kα radiation. Size-exclusion chromatography (SEC) measurements were performed at 40 °C on a Shodex model GPC-101 system with two polystyrene gel columns in series (Shodex KF-806M) and equipped with a refractive index (RI) detector. The mobile phase was tetrahydrofuran (THF) at a flow rate of 1.0 mL/min. Scanning electron microscopy (SEM) measurements were performed using a Hitachi model S-3000N at an accelerating voltage of 5 kV. Samples were deposited on a conducting carbon tape attached on a SEM sample holder, then coated with platinum. Particle size distribution analysis was carried out using HORIBA model laser scattering particle size analyzer Partica LA-950. The samples were dispersed in acetone at 25 °C.

2. Materials

All reagents and chemicals used in this study were obtained from Merck KGaA, FUJIFILM Wako Pure Chemicals, and Tokyo Chemical Industry, unless otherwise noted. Deuterated solvents for NMR spectroscopy were purchased from Cambridge Isotope Laboratories. Monodisperse PEGs with

molecular weights (MWs) of 0.20, 0.28, 0.38, 0.55, 0.90, 1.4, 2.0, 4.3, 10.6, and 21.1 kg/mol, denoted as 0.2k, 0.3k, 0.4k, 0.6k, 1k, 1.5k, 2k, 4.5k and 20k, respectively, were purchased from FUJIFILM Wako Pure Chemicals, Sigma-Aldrich, and Alfa Aesar, and used without further purification. The molecular weights of the PEGs were determined by size-exclusion chromatography (SEC) calibrated with PEG standards (Polyethylene glycol Standard ReadyCal Set Mp 102-40,000 for GPC, Sigma-Aldrich) using tetrahydrofuran (THF) as an eluent (Table S1).

Synthesis of 1. $[Zn_2(1,4\text{-benzenedicarboxylate})_2\text{triethylenediamine}]_n$ (**1**) was synthesized according to the previously reported procedure with a slight modification.[S1] *N*,*N*-Dimethylformamide (DMF) (320 mL) solution of $Zn(NO_3)_2 \cdot 4H_2O$ (15.7 g, 60.0 mmol) was added a DMF solution (320 mL) of 1,4-benzenedicarboxylic acid (10.0 g, 61.0 mmol) and triethylenediamine (3.4 g, 30 mmol). The mixture was stirred rigorously at 120 °C for 48 h. The reaction mixture was then cooled down to room temperature and the product was collected by suction filtration. The white powder thus collected was washed by dehydrated DMF (ca. 300 mL) on the funnel to give powdery **1** (33.3 g) with DMF as the guest solvent. DMF. The powder X-ray diffraction (PXRD) pattern of **1** was consistent with the simulated patterns previously published (Figure S1).[S1] **1** was evacuated under vacuum at 120 ℃ for 16 h prior to use for the adsorption experiments. SEM images for **1** showed typical morphologies of crystalline particles with particle sizes ranging from approximately 3 to 10 μm (Figures S2).

Synthesis of 2. $[Zn_2(1,4\text{-naphthalenedicarboxylate})_2\text{triethylenediamine}]_n$ (**2**) was synthesized according to the previously reported procedure with a slight modification.[S2] *N*,*N*-Dimethylformamide (DMF) (320 mL) solution of $Zn(NO_3)_2 \cdot 6H_2O$ (16.0 g, 53.8 mmol) was added a DMF solution (320 mL) of 1,4-naphthalenedicarboxylic acid (11.6 g, 53.7 mmol) and triethylenediamine (2.99 g, 26.7 mmol). The mixture was stirred rigorously at 120 °C for 48 h. The reaction mixture was then cooled down to room temperature and the product was collected by suction filtration. The white powder thus collected was washed by dehydrated DMF (ca. 300 mL) on the funnel to give powdery **2** (36.3 g) with DMF as the guest solvent. DMF. The powder X-ray diffraction (PXRD) pattern of **1** was consistent with the simulated patterns previously published (Figure S1).[S2] **2** was evacuated under vacuum at 120 ℃ for 16 h prior to use for the adsorption experiments. SEM images for **2** showed typical morphologies of crystalline particles with particle sizes ranging from approximately 5 to 80 μm (Figures S2 and S3).

3. 2D HETCOR NMR Analysis

^{1}H-^{13}C heteronuclear correlation (HETCOR) with frequency-switched Lee-Goldburg (FSLG) homonuclear decoupling was performed at 20 °C on a 9.4 T solid-state NMR spectrometer (Bruker Avance III 400 MHz) equipped with a double resonance (4 mm and 7 mm) magic-angle spinning probe. The HETCOR spectrum was obtained using a recycle delay of 2 s with a spinning rate of 10 kHz. FSLG contact time of ^{1}H-^{13}C cross-polarization was 2 ms.

4. Adsorption Isotherm Measurements

A PEG sample was dissolved in the given solvent (5.0 mL) at designated concentrations ranging from 0.20 to 6.0 mg/g in a glass vial. To each vial was then gently added the vacuum dried MOF (20.0 mg). The vials were left to stand at 40 °C in a thermostated chamber for 48 h to equilibrate. A small aliquot of the supernatant solution was taken from each vial and the solvent was removed under dynamic vacuum. The residual PEG from each vial was then analyzed by ^{1}H NMR spectroscopy using an external standard method (see Section 7). The amount of adsorbed PEG normalized with the MOF unit weight was calculated for each respective concentration to obtain an adsorption isotherm. The MW dependence of the amount of adsorbed PEG was investigated by means of the same protocol using EtOH, DMF, and $CHCl_3$ solutions of PEG 2k (2.0 mg/g) at 40 °C. For experiments conducted over a wide range of MW (0.2k-20k) to identify the effect of different solvents, an equilibration time of 128 h was applied to ensure the fully equilibrated state for all samples.

5. Adsorption Kinetics Measurements

For single component adsorption kinetics measurements, an ethanol solution (50.0 g, 63.4 mL) of PEG (either 2k or 20k) in a glass vial was placed in an oil bath thermostated at the given temperature (40 or 60 °C) and stirred vigorously at a constant stirring rate. To the solution was gently added vacuum dried MOF (190 mg). A small aliquot of the supernatant was removed from the vial at a given time interval after the MOF addition. The adsorbed amount of PEG normalized with the MOF unit weight was determined in the same method using ^{1}H NMR spectroscopy (see Section 7).

A slightly modified procedure was used for competitive adsorption kinetics measurements for a binary mixture of PEGs. An ethanol solution (50.0 g, 63.4 mL) of two PEGs of 2k and 20k (1.2 mg/g each, 2.4 mg/g total) was placed in an oil bath thermostated at 40 °C and stirred vigorously at a constant stirring rate. To the solution was gently added vacuum dried MOF (190 mg). A small aliquot of the supernatant was removed from the vial at a given time interval after the MOF addition. The total amount of adsorbed PEGs normalized with the MOF unit weight was determined in the same method described above using ^{1}H NMR spectroscopy. The composition ratio of adsorbed PEGs was determined separately by means of SEC (See Section 7).

6. HPLC Analysis on MOF-Packed Column

Preparation of a **2**-packed column is described briefly.[S3] Briefly, powdery crystals of **2** (0.80 g, particle diameter: 5-80 μm, Figure S3) were added to a stainless-steel column (I.D. = 4 mm, L = 250 mm; GL Sciences Inc. model 6010-11045) and filled by a tapping method. The **2**-packed column was connected to a Shimadzu HPLC Prominence system equipped with an evaporative light scattering detector (ELSD) (Shimadzu ELSD-LTII). GPC-grade DMF (FUJIFILM Wako Pure Chemicals) was used as a mobile phase at a flow rate of 1.0 mL/min and column pressure of ~1.4 MPa. The PEG samples were dissolved in DMF at 1 mg/mL concentration and a 5 μL aliquot was injected into the column. The chromatograms were corrected by reference to the column hold-up time (V_0) determined using the elution peak of 1,3,5-triphenylbenzene, which is large enough compared to the pore size of MOFs not

to show any appreciable adsorption.[S4] The corrected retention volume (V_c) for each PEG was thus determined as $V_c = V - V_0$, where V is the observed retention volume.

7. General Procedure for Determination of Adsorbed Amount of PEG

For Single-Component System. The adsorption amount of PEG into the MOF phase was determined by quantification of the PEG concentration of the supernatant solution phase. The amount of PEG adsorbed in a unit weight (1 g) of MOF (m_{ad}) is described as,

$$m_{ad} = \frac{(c_0 - c_t)m_1}{m_2},$$

where c_0 is the initial concentration of the PEG solution used, c_t is the PEG concentration of supernatant solution after given adsorption time, m_1 is the amount of the PEG solution, and m_2 is the amount of MOF added in the solution. c_t was quantified by using ^{1}H NMR as follows. A negligibly small aliquot of the supernatant solution was taken from the parent suspension into a glass vial. After thorough removal of the solvent in the vial, the remaining solid residue (PEG) was dissolved in deuterated chloroform with an external reference. As the external reference, toluene or DMSO was used. The concentration of the reference compound was fixed at 50 μL per 30 mL of deuterated chloroform (concentration, c^*), thus allowing quantification of PEG amount in the residue of the supernatant solution by using ^{1}H NMR analysis. When removing supernatant solution from the sample vial, a small loss of solvent via evaporation into the atmosphere was unavoidable. This may cause a possible error in this experiment, which could be seen as the negative plots in adsorption isotherms for Class 2 solvents (Figure 2).

Based on the integration areas of proton signals for PEG (methylene protons) and the reference compound, c_t was calculated by following equation,

$$c_t = \frac{1}{m_3}\left(\frac{m_4 \times \frac{Rc^*}{R_0}}{1 - \frac{Rc^*}{R_0}}\right),$$

where R_0 is the peak area ratio between PEG and the reference compound of the initial solution used, R is the peak area ratio between PEG and the reference compound after given adsorption time, m_3 is the weight of the supernatant sample taken from the parent suspension, and m_4 is the weight of the deuterated chloroform added to the vial in order to perform ^{1}H NMR analysis.

For Double-Component System. The total amount of adsorbed PEGs, 2k and 20k, in the MOF phase was determined by quantification of the total PEG concentration of the supernatant solution phase. The protocol is the same as that used for the single-component system. Separately, the composition ratio between PEG 2k and 20k adsorbed in the MOF phase was determined by using SEC equipped with an RI detector. In general, the RI signal intensity (I) on the chromatogram is described as,

$$I = kc\frac{dn}{dc},$$

where k is an apparatus constant, c is sample concentration, and dn/dc is RI increment of the present

system. Therefore, the ratio of RI peak area between PEG 2k and 20k on the SEC chromatogram is proportional to the composition ratio between PEG 2k and 20k. Owing to this proportional relationship, the composition ratio of the adsorbed PEGs was determined based on the peak area analysis on the SEC chromatogram of the supernatant solution phase. The adsorption amount of each PEG was thus calculated using both total adsorption amount and composition information obtained via ^{1}H NMR and SEC analyses, respectively.

8. Calculation of Effective Diffusion Coefficient

In this study, following pseudo-second-order equation was used to analyze the adsorption kinetics into the MOF,

$$\frac{dq(t)}{dt} = k\{q(t) - q_e\}^2,$$

where k is the pseudo-second-order rate constant, $q(t)$ is the mount of adsorbed PEG after time t, and q_e is the maximum adsorption amount of PEG at the given concentration at the equilibrated state.[S5] We calculated the effective diffusion coefficient, D_{eff}, which can be related to the rate constant k as follows. For the present PEG adsorption into **2**, we assume Fickian diffusion in the 1D channel with the length of $2L$. Following general Fick's second law was used,

$$\frac{\partial c(x,t)}{\partial t} = D_{\text{eff}} \frac{\partial^2 c(x,t)}{\partial x^2},$$

where $c(x,t)$ is local PEG concentration at specific location x and time t. The initial mole concentration of PEG (defined as mole number of the repeating unit of PEG chain in a unit cell volume) in the channel is 0, namely $c(x,t) = 0$ $(x \neq 0, 2L)$. The boundary condition at the aperture surface (001 face) is $c(0,t) = c(2L,t) = c_0$, where c_0 is the mole concentration of PEG (defined as mole number of the repeating unit of PEG chain in a unit cell volume) at the MOF/solution interface. Here we assume that PEG molecules are adsorbed on the MOF surface with very large equilibrium constant, *K*, in the first step, then diffuse into the 1D MOF channels. Meanwhile, PEGs are continuously supplied from the solution phase to the adsorption layer on the MOF surface. Therefore, c_0 always equals to the maximum loading amount of PEG (0.3 g g^{-1}, which corresponds to c_0 = 4.58) into **2** under the current assumption. The amount of PEG adsorbed into the channel at time t, which is denoted by X, is given by,

$$X = \int_0^{2L} c(x,t)dx = 2c_0L - \frac{4c_0L}{\pi^2}\sum_{n=1}^{\infty}\left(\frac{\cos(n\pi) - 1}{n}\right)^2 \exp\left(-D\left(\frac{n\pi}{2L}\right)^2 t\right).$$

In this consideration, the MOF particles consist of single crystals without channel defect. Therefore, the channel length, $2L$, corresponds to the particle diameter. For the calculation of D_{eff}, actual particle size distribution of **2**, which was measured by laser light scattering method (Figure S3), was used. Taking the size distribution into account, the temporal changes of the adsorbed amount, X, were calculated by iterative numerical calculations. The results gave the relationship between diffusion coefficient (D [m^2 s^{-1}]) and the rate constant (k [g^{-1} min^{-1}]), which is expressed by

$$D_{\text{eff}} = 2.364 \times 10^{-13}\ k.$$

The above equation was applied to determine D_{eff} values for the PEG 2k and 20k insertion into **2**.

It should be noted that we assume that the PEG diffusion occurs only along *c*-axis of the MOF for this calculation. This assumption is supported by our previous simulation study of molten PEG (M_w = 650) insertion into **1**, which shows that the PEG diffusion predominantly occurs along *c*-axis whereas the diffusion along *a*- and *b*-axes is strongly impeded by the narrower window size on *ac* and *bc* planes[S6]

9. van't Hoff Plot

The thermodynamic parameters for the PEG adsorption process were determined by van't Hoff plot analyses on the chromatographic data. The chromatograms for each PEG (0.2k-2k) on the **2**-packed column were obtained at different column temperatures ranging from 30 °C to 75°C. The evaporative light scattering detector (ELSD) signal was recorded with a data sampling rate of 2 Hz. Elution peak analysis for all chromatograms was performed using Origin 8.5.0J SR1 software. A typical FFT filter was applied for smoothing the trace to determine V_c at the peak top position precisely. Following FFT parameters were used. For PEG 0.2k-1k, points of window = 200, Cutoff frequency = 0.125 Hz. For PEG 1.5k and 2k, points of window = 500, Cutoff frequency = 0.0500 Hz. The van't Hoff plot thus obtained for each PEG is given in Figure S9.

10. Supporting Table

Table S1. Molecular weights of PEGs measured by SEC.

Code	M_n	M_w	M_w/M_n
**0.2k**	200	220	1.07
**0.3k**	280	310	1.08
**0.4k**	380	410	1.07
**0.6k**	540	580	1.06
**1k**	910	960	1.05
**1.5k**	1,410	1,480	1.05
**2k**	1,960	2,030	1.04
**4.5k**	4290	4530	1.05
**20k**	21,000	23,600	1.11

11. Supporting Figures

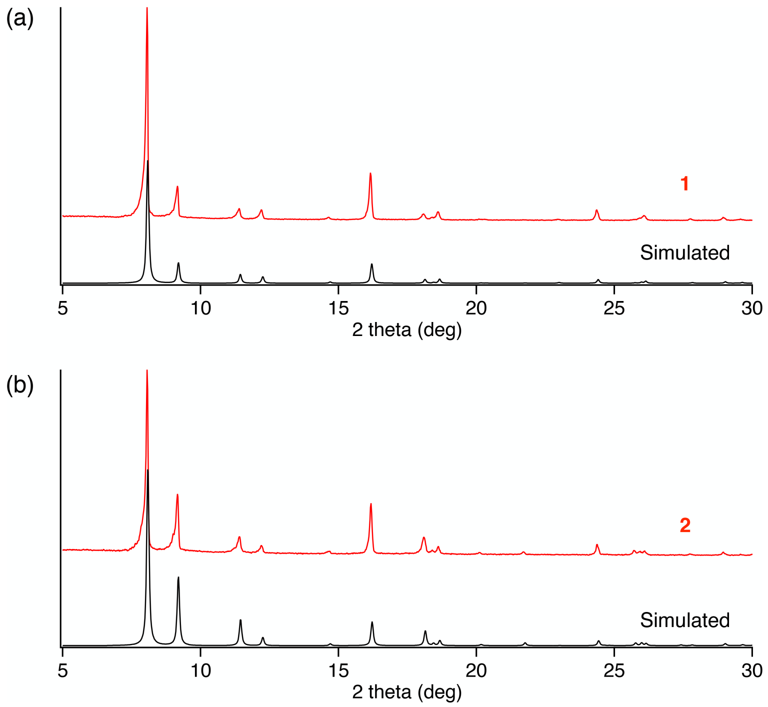

Figure S1. PXRD data of synthesized (a) **1** and (b) **2** after evacuation. Black lines represent the simulated PXRD patterns for respective MOFs without guest molecules.[S1,S2]

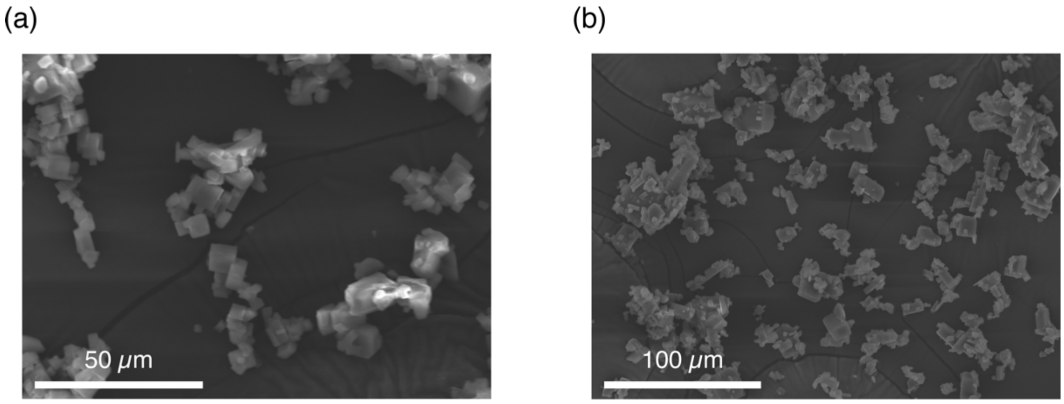

Figure S2. SEM images of (a) **1** and (b) **2** used in this study. The particle size is ranging from 3 to 10 μm for **1** and from 5 to 80 μm for **2**.

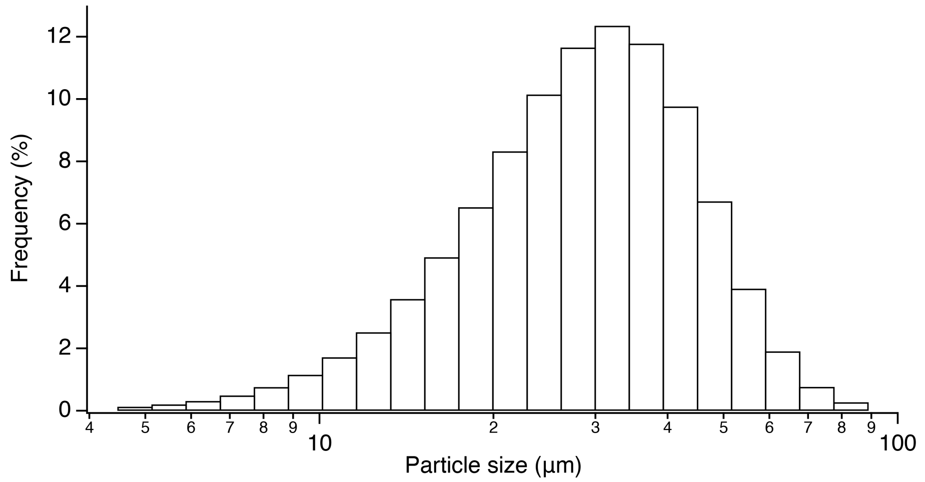

Figure S3. Particle size distribution of **2** measured by laser scattering particle size analyzer.

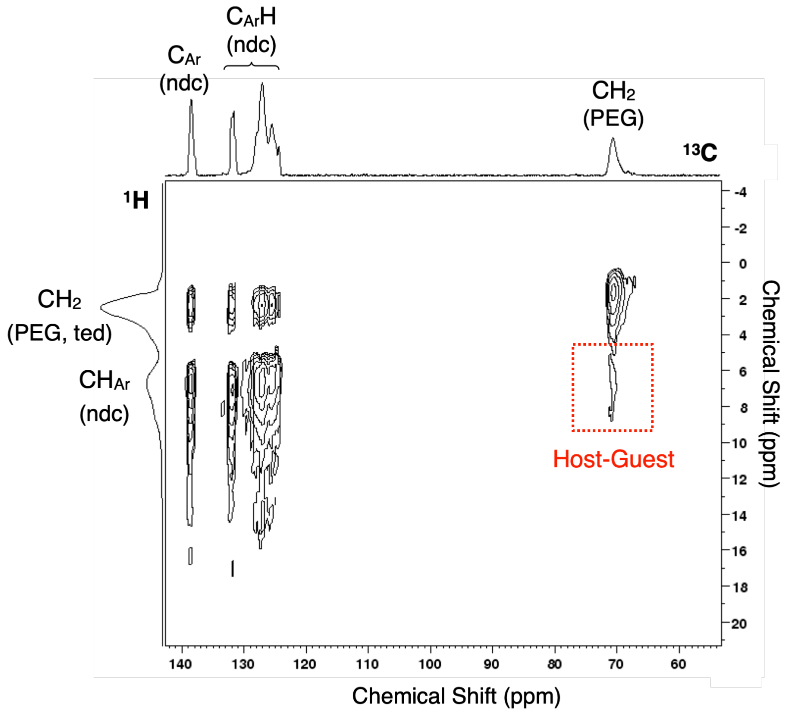

Figure S4. Solid-state 2D ^{1}H-^{13}C HETCOR NMR spectrum of **2**⊃PEG 20k obtained by the liquid-phase adsorption experiment using EtOH as the solvent.

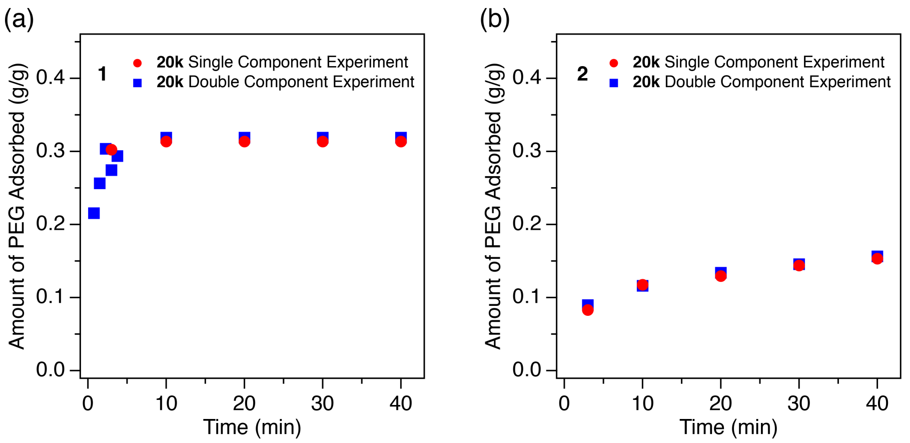

Figure S5. Kinetic curves for PEG 20k measured at 40 °C in (red circle) single-component and (blue square) double-component experiments for (a) **1** and (b) **2**, replotted from Figures 5 and 6. Identical PEG insertion kinetics was observed for PEG 20k in single and double component adsorption experiments. This suggests that PEG 20k insertion is the speed limiting process and not likely affected by presence/absence of the competitor, PEG 2k.

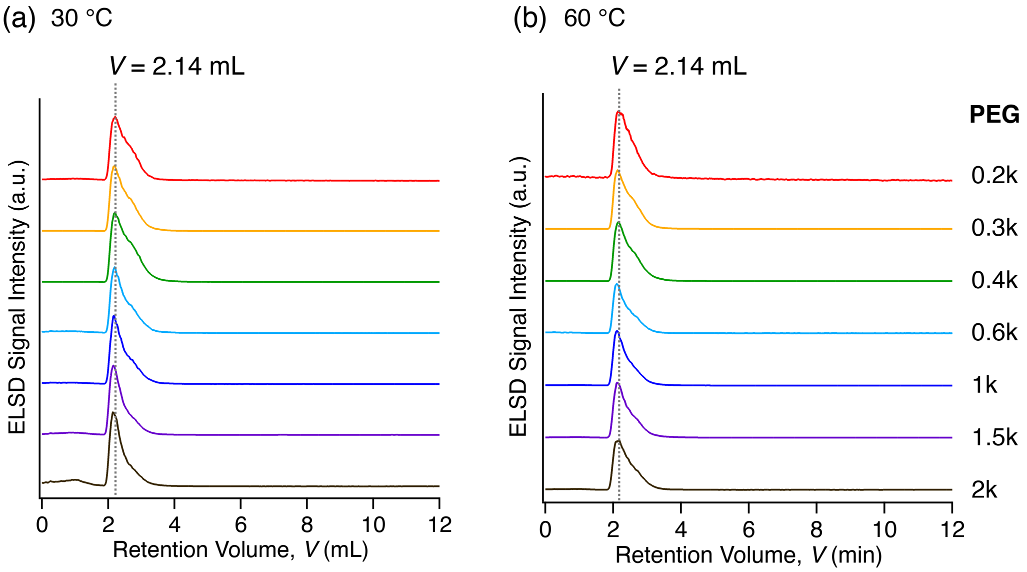

Figure S6. HPLC chromatograms on the **1**-packed column for PEGs with the MW of 0.2k to 2k, measured at (a) 30 °C and (b) 60 °C. No appreciable column retention was observed as all the PEGs are eluted at the same retention volume regardless of the temperature change. The **1**-packed column was prepared according to the literature procedure.[S3] Briefly, powdery crystals of **1** (1.10 g, particle diameter: 3-10 μm) was filled in a stainless-steel column (I.D. = 4 mm, L = 250 mm; GL Sciences Inc. model 6010-11045) by a tapping method. The analytical procedure employed was the same as that for the **2**-packed column, described in the main text.

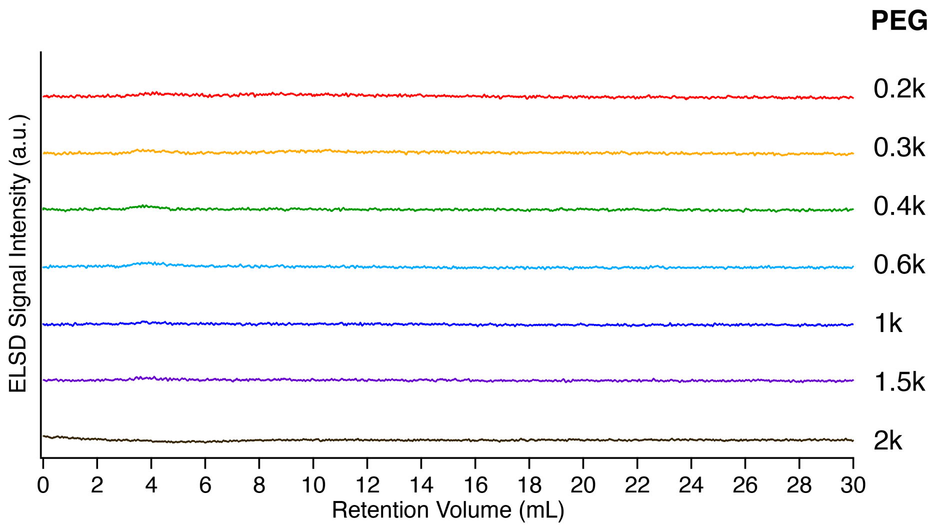

Figure S7. HPLC chromatograms for PEGs on the **2**-packed column using EtOH as the eluent at 60 °C.

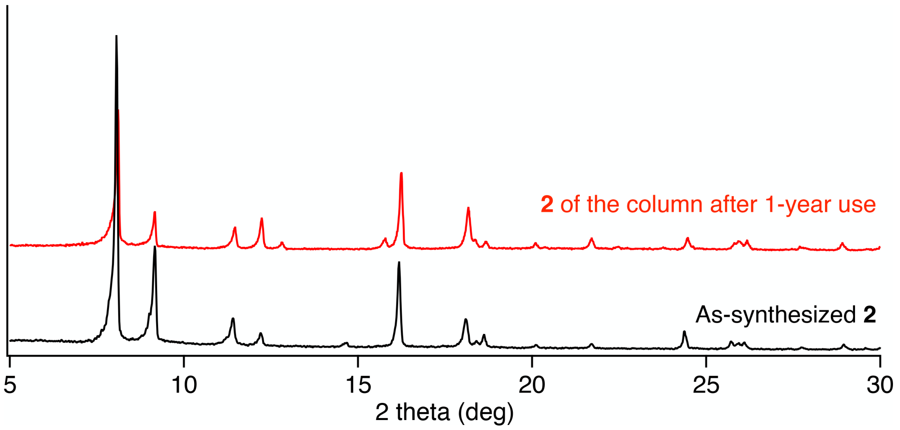

Figure S8. PXRD data of (black) as-synthesized **2** including DMF as the guest solvent and (red) **2** recovered from the **2**-packed column used with DMF at 80 °C for 1 year.

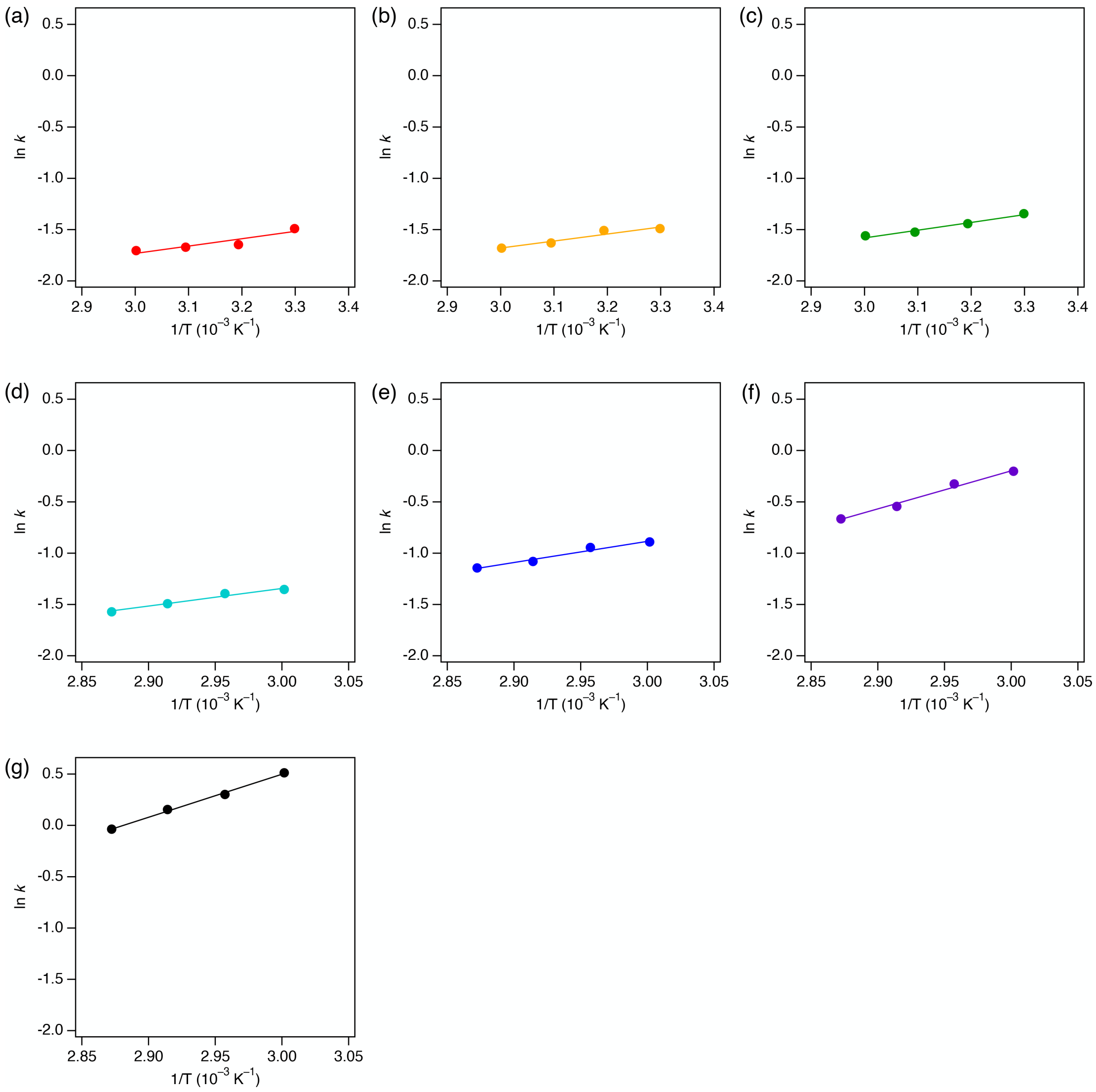

Figure S9. van't Hoff plots for PEG (a) 0.2k, (b) 0.3k, (c) 0.4k, (d) 0.6k, (e) 1k, (f) 1.5k, and (g) 2k.

12. Supporting References

(S1) D. N. Dybtsev, H. Chun, K. Kim, *Angew. Chem. Int. Ed.*, 2004, **43**, 5033–5036.

(S2) H. Chun, D. N. Dybtsev, H. Kim, K. Kim, *Chem. Eur. J.*, 2005, **11**, 3521–3529.

(S3) N. Mizutani, N. Hosono, B. Le Ouay, T. Kitao, R. Matsuura, T. Kubo, T. Uemura, *J. Am. Chem. Soc.*, 2020, **142**, 3701–3705.

(S4) N. Hosono, A. Terashima, S. Kusaka, R. Matsuda, S. Kitagawa, *Nat. Chem.*, 2019, **11**, 109–116.

(S5) Y. S. Ho, G. McKay, *Process Biochem.*, 1999, **34**, 451–465.

(S6) T. Uemura, G. Washino, S. Kitagawa, H. Takahashi, A. Yoshida, K. Takeyasu, M. Takayanagi and M. Nagaoka, *J. Phys. Chem. C*, **2015**, *119*, 21504–21514.
